# Acute cholecystitis secondary to dog bite

**DOI:** 10.1016/j.ijscr.2019.02.012

**Published:** 2019-02-12

**Authors:** James Elliott, Eric Donaldson

**Affiliations:** Department of General Surgery, Toowoomba Hospital, 154 Pechey Street, South Toowoomba, Queensland, Australia

## Abstract

**Introduction:**

The gram-negative bacteria known as Capnocytophaga canimorsus (C. canimorsus) is found in dog saliva and rarely can cause severe infection in humans following a bite or scratch. There has previously been just a single case described in the literature of Acute Acalculous Cholecystitis (AAC) secondary to C. canimorsus.

**Presentation of case:**

Here we describe the second ever published case of C. canimorsus bacteremia presenting with acute cholecystitis. The patient presented with epigastric pain and sepsis three weeks post domestic dog bite. On further examination, he was Murphy’s sign positive. Investigations included blood cultures, Ultrasound Scan and Computed Tomography of the Abdomen. He was treated with intravenous fluid resuscitation, and intravenous ceftriaxone and metronidazole. He required an extended course of antibiotics for complete symptom resolution. His blood cultures were positive for C. Canimorsus.

**Discussion:**

This case highlights the ever-present need for thorough history and examination, and consideration of prolonged antibiotics for cases of cholecystitis that could be secondary to C. canimorsus bacteremia.

**Conclusion:**

We strongly advocate blood cultures in patients who present with abdominal pain and sepsis, particularly when they have a recent history of animal bite. In cases of cholecystitis secondary to C. canimorsus it may be necessary to monitor the patient’s progress more closely and treat with prolonged targeted antibiotic therapy.

## Highlights

• This is the second known case of Cholecystitis due to Capnocytophaga canimorsus.
• Accurate diagnosis of this condition requires thorough history and examination.
• Prolonged targeted antibiotic therapy may be required for symptom resolution.

## 1 Introduction

The gram-negative bacteria known as Capnocytophaga canimorsus (C. canimorsus) is found in dog saliva and rarely can cause severe infection in humans following a bite or scratch [1]. Infection typically only affects immunocompromised hosts [1]. Main risk factors for infection with C. canimorsus include asplenia, functional asplenia, cirrhosis, and a history of alcohol dependence [2]. A range of patient cases with other immunocompromised states have been described [[3], [4], [5], [6]]. The small percentage of patients who become unwell usually present at an average of five to six days post bite [7] with one or a combination of the following: septic shock (41%), fever of unknown origin (13%), meningitis (13%), cellulitis (11%), and respiratory tract infection (7%) [2]. There are also case reports of endocarditis, septic arthritis, and purpura fulminans [2,8]. There has been just a single case described in the literature of acute acalculous cholecystitis secondary to C. canimorsus [9]. This was in an octogenarian female with a history of hypertension and diabetes mellitus.

Acalculous cholecystitis is an inflammatory disease process of the gallbladder and is thought to result from factors that promote gallbladder stasis and ischaemia. Such risk factors are wide-ranging, but there are recognised primary infections that are associated with acalculous cholecystitis. These include but are not limited to: Ascaris lumbricoides, Brucella species, Campylobacter jejuni, Candida species, Coxiella burnetii, Cryptosporidium, Cytomegalovirus, Echinococcus granulosus, Epstein-Barr virus, Flavivirus, Hepatitis A and B, Isospora, Leptospira species, Mycobacterium tuberculosis, Plasmodium species, Salmonella species and Vibrio cholerae [10,11]. Acalculous cholecystitis is more characteristically secondary to critical illness in already-hospitalised patients, though it is probably under-recognised in the outpatient setting [12].

This case has been reported in line with the SCARE criteria [13].

## 2 Presentation of case

**Fig. 1:**
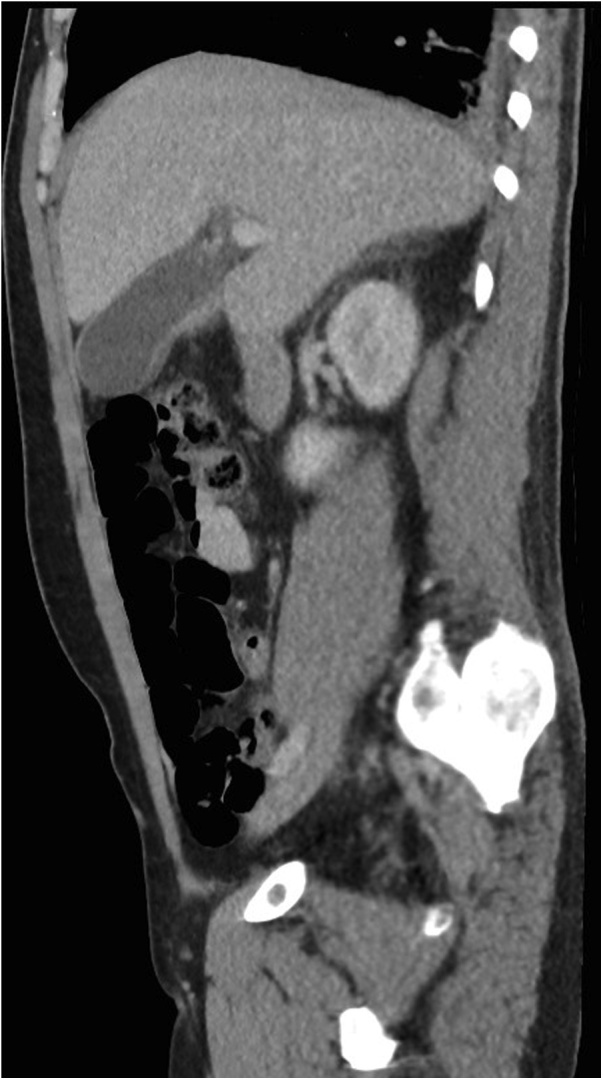
Oedematous gallbladder suggestive of cholecystitis with no evidence of calculi.

Here we describe the second ever published case of C. canimorsus bacteremia presenting with acute cholecystitis, albeit in a very different host: a forty year old Caucasian male tradesperson, with normal BMI, no medical conditions but previous heavy alcohol use and current cigarette smoking. The patient presented by ambulance to our institution with epigastric pain and sepsis three weeks post domestic dog bite to his right lower leg. A healing superficial bite wound was still present and healing well and with no signs of local infection. On examination, his Murphy’s sign was positive. Investigations included blood cultures and CT-Abdomen (Fig. 1). CT revealed a distended and oedematous gallbladder suggestive of cholecystitis and he was treated with intravenous fluid resuscitation, and intravenous ceftriaxone and metronidazole. His blood cultures were positive for C. Canimorsus. Subsequent abdominal ultrasound imaging confirmed acalculous cholecystitis without evidence of perforation or gangrene: both recognised complications [[14], [15], [16]]. He improved rapidly with aggressive conservative treatment, and after seventy two hours of intravenous antibiotic therapy his treatment was converted to oral oral amoxicillin with clavulanic acid. He required a prolonged course of this (four weeks) for complete symptom resolution. The patient thus avoided emergency drainage or cholecystectomy. He has had no recurrent symptoms.

## 3 Discussion

This case highlights the ever-present need for thorough history and examination of a patient presenting with septic shock, as his dog bite was not obviously related at first. Blood cultures and abdominal imaging are essential in patients with a febrile illness, right upper quadrant pain, and distributive shock. Based on this case, we would encourage consideration of prolonged antibiotics in cases of acalculous cholecystitis that could be secondary to c. canimorsus bacteremia. Given the risk of recurrence of this illness is very low, interval cholecystectomy is considered unnecessary in patients who recovery from acute acalculous cholecystitis [17].

## 4 Conclusion

Acute Cholecystitis secondary to C. Canimorsus bacteremia is rare but still likely under-recognised. The bacteria may have enough pathogenicity to cause cholecystitis in hosts who are not immunodeficient such as this patient. We strongly advocate blood cultures in patients who present with abdominal pain and sepsis, particularly when they have a recent history of bite. In cases of cholecystitis secondary to C. Canimorsus it may be necessary to monitor the patient closely and treat with prolonged targeted antibiotic therapy.

## Conflict of interest

Nothing to declare.

## Sources of funding

Nothing to declare.

## Ethical approval

This study has been granted status of “Not Requiring Ethical Review” (NRER) by the local HREC (Darling Downs Hospital and Health Service). Reference: LNR/2019/QTDD/51019 (Jan ver 1).

## Consent

Written informed consent was obtained from the patient for publication of this case report and any accompanying de-identified images. A copy of the written consent is available for review by the Editor-in-Chief of this journal on request.

## Author contribution

Dr James Elliott is the primary author of this case report, and authored the entire paper and associated documentation.

Dr Eric Donaldson was the patient’s treating consultant during their hospital admission, and his role in this paper was to supervise the drafting and writing of this case report paper.

## Registration of research studies

Since this is a case report, not a research study, registration is not indicated.

## Guarantor

Dr James Elliott (the primary author) is the guarantor of this work.

## Provenance and peer review

Commissioned, externally peer-reviewed.
